# Comments on Li *et al*. Effects of *in Utero* Exposure to Dicyclohexyl Phthalate on Rat Fetal Leydig Cells. *Int. J. Environ. Res. Public Health* 2016, *13*, 246

**DOI:** 10.3390/ijerph13060532

**Published:** 2016-05-25

**Authors:** Terje Svingen

**Affiliations:** Division of Diet, Disease Prevention and Toxicology, National Food Institute, Technical University of Denmark, Søborg DK-2860, Denmark; tesv@food.dtu.dk; Tel.: +45-3588-7532

## Abstract

Profiling the expression levels of genes or proteins in tissues comprising two or more cell types is commonplace in biological sciences. Such analyses present particular challenges, however, for example a potential shift in cellular composition, or ‘cellularity’, between specimens. That is, does an observed change in expression level represent what occurs within individual cells, or does it represent a shift in the ratio of different cell types within the tissue? This commentary attempts to highlight the importance of considering cellularity when interpreting quantitative expression data, using the mammalian testis and a recent study on the effects of phthalate exposure on testis function as an example.

All tissues comprising two or more cell types are subject to a shift in cellularity. In turn, a change in tissue cellularity can significantly influence expression level read-outs. This issue is frequently ignored, or at least not mentioned, when interpreting quantitative expression data. Consequently, important information may be overlooked or lead to erroneous conclusions. Taking cellularity into consideration may in some instances also allow for alternative interpretations of the data. To further illustrate this point this commentary, using the testis as an example, draws from a recent study [1] published in the March issue of this journal to highlight the importance of considering tissue cellularity. 

In their manuscript [1], Li *et al*. report that *in utero* exposure to dicyclohexyl phthalate (DCHP) affect rat fetal Leydig cells and their ability to synthesise vital hormones. This is an interesting study offering some new insight into how phthalates interfere with fetal testis development and function. The study joins a significant number of other studies reporting on similar effects of phthalates—namely reduced testosterone and insulin-like 3 (INSL3) expression and the formation of Leydig cell aggregates resulting in phenotypic manifestations in androgen- and INSL3-responsive tissues—often collectively referred to as the “phthalate syndrome” [2]. The fact that DCHP exposure can affect male reproductive development has been known for some time, but Li and co-workers also wanted to elucidate further its direct effect on fetal Leydig cells. Based on experimental data, one of the conclusions drawn was that “*in utero* exposure to DCHP affects the expression levels of fetal Leydig cell steroidogenic genes”. This may be so, but considering testicular cellularity after phthalate exposure, the data may also allow for alternative interpretations. 

The mammalian testis is a complex organ comprising more than ten different cell types from early development [3]. Thus, cellularity has been highlighted as an important parameter to consider when quantifying expression levels in the testis [4,5] and is sometimes explicitly addressed [6,7,8,9]. The fact is that altered quantitative expression data from multicellular tissues may represent either (i) a *bona fide* change in expression levels within individual cells, or (ii) reflect a shift in the ratio between different cell types. Fortunately, Li and co-workers carefully characterised the histopathological consequence of DCHP exposure, which revealed an altered Leydig cell morphology, but unaltered Leydig cell numbers [1]. Based on the latter observation, one could infer that testicular cellularity was relatively unchanged, at least pertaining to the Leydig cells and thus, that the lower expression of Leydig cell-specific steroidogenic genes was due to downregulation of gene transcription. Based on the former observation, however, a significant shift in cellularity may have occurred and transcription potentially not affected *per se*. 

The genes assessed by RT-qPCR were for the most part downregulated in the higher dosed animals, or unchanged as in the case of Cyp11a1. Other Leydig cell-specific genes such as Insl3, Star and Hsd3b1 were significantly suppressed and notably, no genes were shown to be upregulated. The fact that Cyp11a1 was the only Leydig cell-specific gene not affected could suggest an alternative mode of action regarding the effects of DCHP on the fetal testis. Since Cyp11a1 is one of the earliest markers expressed by immature Leydig cells, its expression without concomitant expression of markers of more mature Leydig cells such as Insl3, could suggest that DCHP exposure causes a loss of mature Leydig cells and that majority of the Leydig cell are more immature. Thus, it would have been interesting to include additional Leydig cell markers in the analysis, not least Delta-like 1 homolog (Dlk1), which has been shown to be a marker for immature Leydig cells, but—as opposed to Cyp11a1—lost in mature, Insl3-expressing Leydig cells [10]. Previous studies have in fact shown abnormal positioning, and perhaps disrupted differentiation, of rat Leydig cells in response to phthalate exposure [11,12], but notably also affecting Cyp11a1 [13]. 

This commentary has by no means been, and was not intended as an exhaustive review of the testis-related molecular effects caused by phthalate exposure. The testis, and the study by Li and co-workers, served chiefly to highlight the importance of taking tissue cellularity into account when inferring biological relevance from quantitative expression data. It is clear from a large body of evidence that phthalates affect testis function and numerous studies have shown a clear reduction in testosterone and INSL3 levels following *in utero* exposure, with subsequent consequences for male reproductive health [14,15]. But whether various phthalates directly dysregulate gene expression or affect Leydig cell differentiation and maintenance more broadly, remains somewhat obscure. Of course, one might argue that it doesn’t really matter if it is gene expression or Leydig cell differentiation that causes a reduced level of testosterone. The end result is still the same; androgen insufficiency and feminised male offspring. Similar arguments could most likely be made for many other tissues under various situations. But arguing that it doesn’t really matter whether curiosity or malevolence killed the cat—the cat remains dead—is somewhat unsatisfactory. And I, for one, can imagine many scenarios where it actually matters deeply.

## References

[1] Li X., Chen X., Hu G., Li L., Su H., Wang Y., Chen D., Zhu Q., Li C., Li J. (2016). Effects of *in utero* exposure to dicyclohexyl phthalate on rat fetal leydig cells. Int. J. Environ. Res. Public Health.

[2] Gray L.E.J., Foster P.M.D. (2003). Significance of experimental studies for assessing adverse effects of endocrine-disrupting chemicals. Pure Appl. Chem..

[3] Svingen T., Koopman P. (2013). Building the mammalian testis: Origins, differentiation, and assembly of the component cell populations. Genes Dev..

[4] Ivell R., Spiess A.N., Rommerts F.F.G., Teerds K.J. (2002). Analysing differential gene expression in the testis. Testicular Tangrams.

[5] Almstrup K., Nielsen J.E., Hansen M.A., Tanaka M., Skakkebaek N.E., Leffers H. (2004). Analysis of cell-type-specific gene expression during mouse spermatogenesis. Biol. Reprod..

[6] Cappallo-Obermann H., Feig C., Schulze W., Spiess A.N. (2013). Fold-change correction values for testicular somatic transcripts in gene expression studies of human spermatogenesis. Hum. Reprod..

[7] Van den Driesche S., Macdonald J., Anderson R.A., Johnston Z.C., Chetty T., Smith L.B., McKinnell C., Dean A., Homer N.Z., Jorgensen A. (2015). Prolonged exposure to acetaminophen reduces testosterone production by the human fetal testis in a xenograft model. Sci. Transl. Med..

[8] Davis B.W., Seabury C.M., Brashear W.A., Li G., Roelke-Parker M., Murphy W.J. (2015). Mechanisms underlying mammalian hybrid sterility in two feline interspecies models. Mol. Biol. Evol..

[9] Svingen T., Jørgensen A., Rajpert-De Meyts E. (2014). Validation of endogenous normalizing genes for expression analyses in adult human testis and germ cell neoplasms. Mol. Hum. Reprod..

[10] Lottrup G., Nielsen J.E., Maroun L.L., Møller L.M., Yassin M., Leffers H., Skakkebæk N.E., Rajpert-De Meyts E. (2014). Expression patterns of dlk1 and insl3 identify stages of leydig cell differentiation during normal development and in testicular pathologies, including testicular cancer and Klinefelter syndrome. Hum. Reprod..

[11] Mahood I.K., McKinnell C., Walker M., Hallmark N., Scott H., Fisher J.S., Rivas A., Hartung S., Ivell R., Mason J.I. (2006). Cellular origins of testicular dysgenesis in rats exposed *in utero* to di(n-butyl) phthalate. Int. J. Androl..

[12] Mahood I.K., Hallmark N., McKinnell C., Walker M., Fisher J.S., Sharpe R.M. (2005). Abnormal leydig cell aggregation in the fetal testis of rats exposed to di(n-butyl) phthalate and its possible role in testicular dysgenesis. Endocrinology.

[13] Hallmark N., Walker M., McKinnell C., Mahood I.K., Scott H., Bayne R., Coutts S., Anderson R.A., Greig I., Morris K. (2007). Effects of monobutyl and di(n-butyl) phthalate *in vitro* on steroidogenesis and leydig cell aggregation in fetal testis explants from the rat: Comparison with effects *in vivo* in the fetal rat and neonatal marmoset and *in vitro* in the human. Environ. Health Perspect..

[14] Sharpe R.M., Skakkebaek N.E. (2008). Testicular dysgenesis syndrome: Mechanistic insights and potential new downstream effects. Fertil. Steril..

[15] Hu G.X., Lian Q.Q., Ge R.S., Hardy D.O., Li X.X. (2009). Phthalate-induced testicular dysgenesis syndrome: Leydig cell influence. Trends Endocrinol. Metab..

